# Enhancing buckwheat maturity classification with generative adversarial networks for spectroscopy data augmentation

**DOI:** 10.3389/fpls.2025.1604088

**Published:** 2025-07-08

**Authors:** Huihui Wang, Xiaoxue Che, Jiaxuan Nan, Yuyuan Miao, Yaqi Wang, Wuping Zhang, Fuzhong Li, Jiwan Han

**Affiliations:** Software College, Shanxi Agricultural University, Taigu, Shanxi, China

**Keywords:** buckwheat, spectroscopy, machine learning, generative adversarial networks, NIR, precision agriculture

## Abstract

**Introduction:**

The optimal harvest period for buckwheat is challenging to determine due to its short growth cycle. Harvesting too early or too late can negatively affect the quality of the crop. Traditional harvest methods are labor-intensive and fail to account for the spatial variability in buckwheat quality within a field. This study explores the use of near-infrared (NIR) spectral data to classify the maturity stages of buckwheat.

**Method:**

Four distinct developmental stages were examined: UM (Unripe Maturity), representing buckwheat harvested at 65 days after sowing; HM (Half Maturity), harvested at 75 days; MS (Full Maturity with Shell), harvested at 85 days with husks intact; and MUS (Full Maturity Unhulled Sample), also harvested at 85 days but manually dehulled. Unlike traditional machine learning models, which require diverse and extensive datasets, this study investigates the use of a conditional WGAN-GP to generate synthetic datasets and improve model performance. Four machine learning models were employed in this study: Support Vector Machine (SVM), Random Forest (RF), k-Nearest Neighbors (KNN), and Partial Least Squares Linear Discriminant Analysis (PLS-LDA).

**Results and Discussion:**

The conditional WGAN with the gradient penalty was trained for a range of epochs: 1000, 2000, 8000, 10,000, and 20,000. After training 10,000 epochs, synthetic hyperspectral reflectance data were very similar to real spectra for each maturity category. To assess the impact of conditional WGAN-GP data augmentation, model performance was first evaluated using the original dataset as a baseline, showing PLS-LDA had the best classification performance with accuracy of 95% and kappa coefficient of 0.93. The models were then trained on a combination of original and synthetic data, revealing that synthetic data can improve the classification model performance for RF and KNN. The best classification performance was achieved by RF with an accuracy of 97% and kappa coefficient of 0.94. This study demonstrates the effectiveness of synthetic data in enhancing classification accuracy.

## 1 Introduction

Buckwheat (*Fagopyrum esculentum*) is a nutritionally valuable pseudocereal cultivated globally for its high-quality protein, starch, fat, flavonoids and phenols (Skřivan et al., 2023). As a gluten-free grain, buckwheat plays a crucial role in functional food development and human nutrition. In addition, buckwheat is an important crop for sustainable agriculture and food security, as its short growth cycle (typically maturing within 70–90 days), allowing for its integration in crop rotation systems to improve soil fertility and reduce pest pressure (Takeshima et al., 2022). Due to the short growth cycle, the determination of buckwheat maturity is critical for optimizing harvest decisions. Traditional buckwheat assessment methods are based on visual inspection and manual classification. In practice, farmers randomly collect samples from the field and assess their external color to determine maturity. However, since the outer husk of buckwheat may not always reflect internal physiological changes, a more detailed evaluation often involves manual dehulling to examine the kernel’s color and hardness. This approach, while widely used, is labor-intensive. In addition, the sample is conducted randomly without accounting spatial variability of soil and microclimatic within field, which may not accurately represent overall maturity distribution of the crop (Lyu et al., 2023). Therefore, there is a pressing need to develop more efficient, scalable, and accurate approaches for assessing buckwheat maturity. Leveraging advanced machine learning and data augmentation techniques to enhance the performance of predictive models represents a promising direction to address these challenges.

To date, researchers have carried out significant work in using proximal and remote sensors to assess buckwheat maturity and quality parameters (Sytar et al., 2017; Yoosefzadeh-Najafabadi et al., 2021; Wu et al., 2022; Yang et al., 2023; Xin et al., 2024). These sensing techniques include hyperspectral and multispectral sensors deployed on satellites and unmanned aerial vehicles (UAVs) for large-scale monitoring, as well as handheld near-infrared spectroscopy (NIRS) devices for detailed, close-range analysis. Hyperspectral, multispectral imaging and NIRS capture reflectance patterns across different wavelengths, enabling the detection of subtle biochemical and structural changes in buckwheat during maturation (Lyu et al., 2024). For example, Xin et al. (2024) explored using NIRS (900–1700 nm) to non-destructive estimate buckwheat maturity and quality parameters. Additionally, Wu et al. (2022) explored using RGB camera deployed on UAV to predict maturity of buckwheat. The results demonstrated that features extracted from RGB images provided reliable performance in predicting the specific growth stage of buckwheat. The integration of these sensing methods provides rapid and scalable solutions for estimating harvest timing.

In traditional spectral data analysis, chemometric techniques such as partial least squares regression (PLSR) and linear discriminant analysis (LDA) have been widely used to extract specific features from high-dimensional spectroscopy data. However, the traditional chemometric techniques can only explore the linear relationship between spectroscopy data and predictions. With the development of machine learning (ML) techniques, support vector machines (SVM), random forests (RF), K-nearest neighbors (KNN) and artificial neural networks (ANN) have shown ability to explore complex non-linear relationship between spectroscopy data and predictions (Vasseur et al., 2022; Xin et al., 2024; Lyu et al., 2025). For example, Lyu et al. (2025) explored using SVM, RF, KNN, and stack ensemble learning model to predict grape quality parameters through NIRS data. However, machine learning (ML) models often face challenges such as overfitting and underfitting. These issues are primarily caused by insufficient or unrepresentative datasets, which fail to cover the full range of variability of the predictions. For instance, Lyu et al. (2025) used 1830 samples to train ML models achieving satisfactory performance by ensuring adequate data coverage. Similarly, Xin et al. (2024) collected 600 near-infrared (NIR) spectral images of buckwheat to train ML models, effectively mitigating overfitting by increasing dataset diversity. These studies highlight the importance of large, diverse, and well-distributed datasets in enhancing model generalization.

In traditional NIRS research, researchers have relied on collecting large numbers of samples to increase dataset size and diversity (Wei et al., 2021; Xin et al., 2024; Lyu et al., 2025). This approach improves model robustness and accuracy but is time-consuming and labor-intensive, requiring extensive field sampling and manual spectral measurements. However, with the advancement of deep learning (DL) techniques, generative adversarial networks (GANs) have shown great potential in synthetic data generation, reducing the dependency on large real-world datasets. In the field of computer vision, GANs have already been widely applied to generate high-quality synthetic images, augmenting datasets for tasks such as object detection and classification (Lu et al., 2022). Inspired by these successes, recent studies have explored the application of GAN-based models in spectral data augmentation, aiming to create realistic synthetic spectra that mimic the distribution of actual NIRS measurements (Jiang et al., 2023). Jiang et al. (2023) compared the performance of different spectral data augmentation methods, including GANs and extended multiplicative signal augmentation (EMSA), in enhancing the prediction accuracy of convolutional neural networks (CNNs) for soil property estimation. The results showed that GANs can generate data very similar to real data and with better diversity. However, their studies primarily focused on unlabeled data, and little attention has been paid to generating labeled hyperspectral reflectance data for multi-class classification tasks, such as crop maturity stage prediction. This gap highlights the need for more advanced generative models capable of producing class-conditional spectral data with high fidelity.

A limitation of conventional GANs architectures is that they can only generate spectral data for a single label at a time. To address this, conditional Wasserstein GAN with Gradient Penalty (WGAN-GP) was developed by Goodfellow et al. (2020) to generate data for multiple labels within a single training process. Compared to traditional GANs, conditional WGAN-GP incorporates an additional classifier, allowing it to generate data with different categories. Compared to other augmentation techniques like EMSA, which apply signal transformations based on predefined rules, conditional WGAN-GP offers a data-driven solution that learns the true data distribution and class-wise differences directly from the training set. This makes it especially suitable for scenarios where spectral data is scarce, and class balance is critical. Conditional WGAN-GP have already demonstrated their potential to generate agricultural RGB and multispectral image, contributing to advancements in crop disease detection and fruit quality assessment (Fawakherji et al., 2020, 2024). However, to the best of the authors’ knowledge, conditional WGAN-GP has not yet been explored for generating spectral data corresponding to different buckwheat maturity stages. Given the critical role of spectral analysis in rapid, non-destructive maturity detection, this study aims to bridge this gap by investigating the feasibility of conditional WGAN-based spectral data augmentation for buckwheat maturity classification.

The key contributions of this study are as follows. First, this research represents the first attempt to apply conditional WGAN-GP for generating labeled near-infrared spectral data corresponding to different buckwheat maturity stages. Second, it systematically evaluates the effectiveness of GAN-based data augmentation in improving the performance of machine learning classifiers for maturity stage prediction. Finally, this study provides insights into data-driven augmentation strategies for addressing class imbalance and dataset limitations in spectral analysis for crop monitoring. The remainder of this thesis is organized as follows. Chapter 2 describes the materials and methods, including data collection, preprocessing, model development, and evaluation metrics. Chapter 3 presents the experimental results and analysis. Chapter 4 presents the discussion of experimental results in relation to prior research and practical implications. Finally, Chapter 5 summarizes the key findings, discusses limitations, and outlines future research directions.

## 2 Method

### 2.1 Buckwheat sample collection

**Figure 1:**
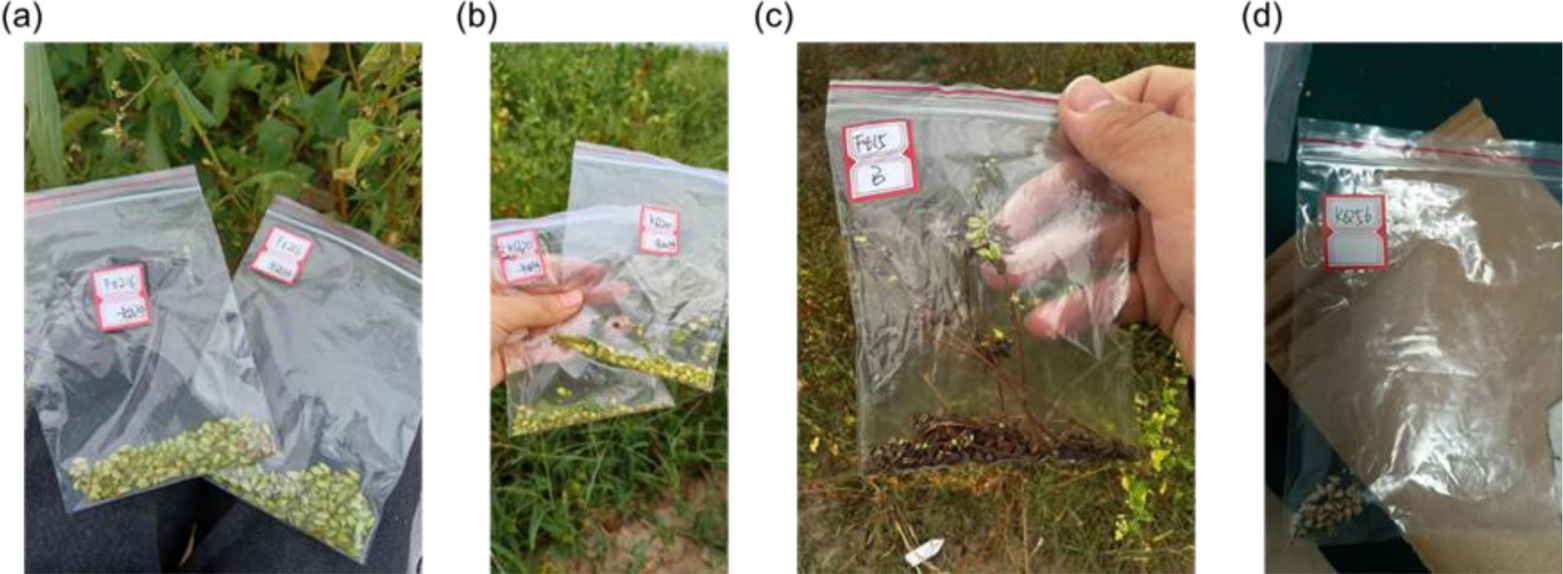
Four maturity stages of buckwheat used in this study including: irrigation period (65 days) **(a)**, green-ripe stage (75 days) **(b)**, harvest stage (85 days) with husks **(c)**, and harvest stage dehulled **(d)**.

The buckwheat samples used in this research were collected from the Tai Gu (37°41′N, 112°58′E) and Ke Lan (38°76′N, 111°62′E) experiment fields at Shanxi Agricultural University, Shanxi Province. The average annual rainfall is 456 mm, and the average temperature is 9.8°C. In experiment fields, the buckwheat was planted in blocks with each block consisting of six rows with 1.5 m length and 30cm apart. There are 708 buckwheat varieties in the experiment fields. The buckwheat was planted on 3rd May 2024 in Tai Gu and 23rd May 2024 in Ke Lan. Field management was carried out in strict accordance with standard agricultural practices, including routine irrigation and weed control to ensure optimally growing conditions. In this study, buckwheat was divided into three harvest periods: the growth cycles were 65 days, 75 days, and 85 days. During each harvest period, the buckwheat was manually collected from fields and brought back to the hyperspectral imaging lab immediately. In this study 146 buckwheat varieties were collected each harvest day. During sampling, 10 buckwheat plants were randomly selected from each block within 146 varieties, and their kernels were collected for further analysis. Notably, buckwheat harvested on day 85 of the growth cycle was fully matured. To facilitate further analysis, the collected kernels were divided into four maturity stages of buckwheat (**Figure 1**). The four maturity stages including: UM, corresponding to samples harvested 65 days after sowing; HM, harvested at 75 days; MS, harvested at 85 days with the husks remaining intact; and MUS, also harvested at 85 days but with the husks manually removed.

### 2.2 Hyperspectral imaging spectroscopy data acquisition

**Figure 2:**
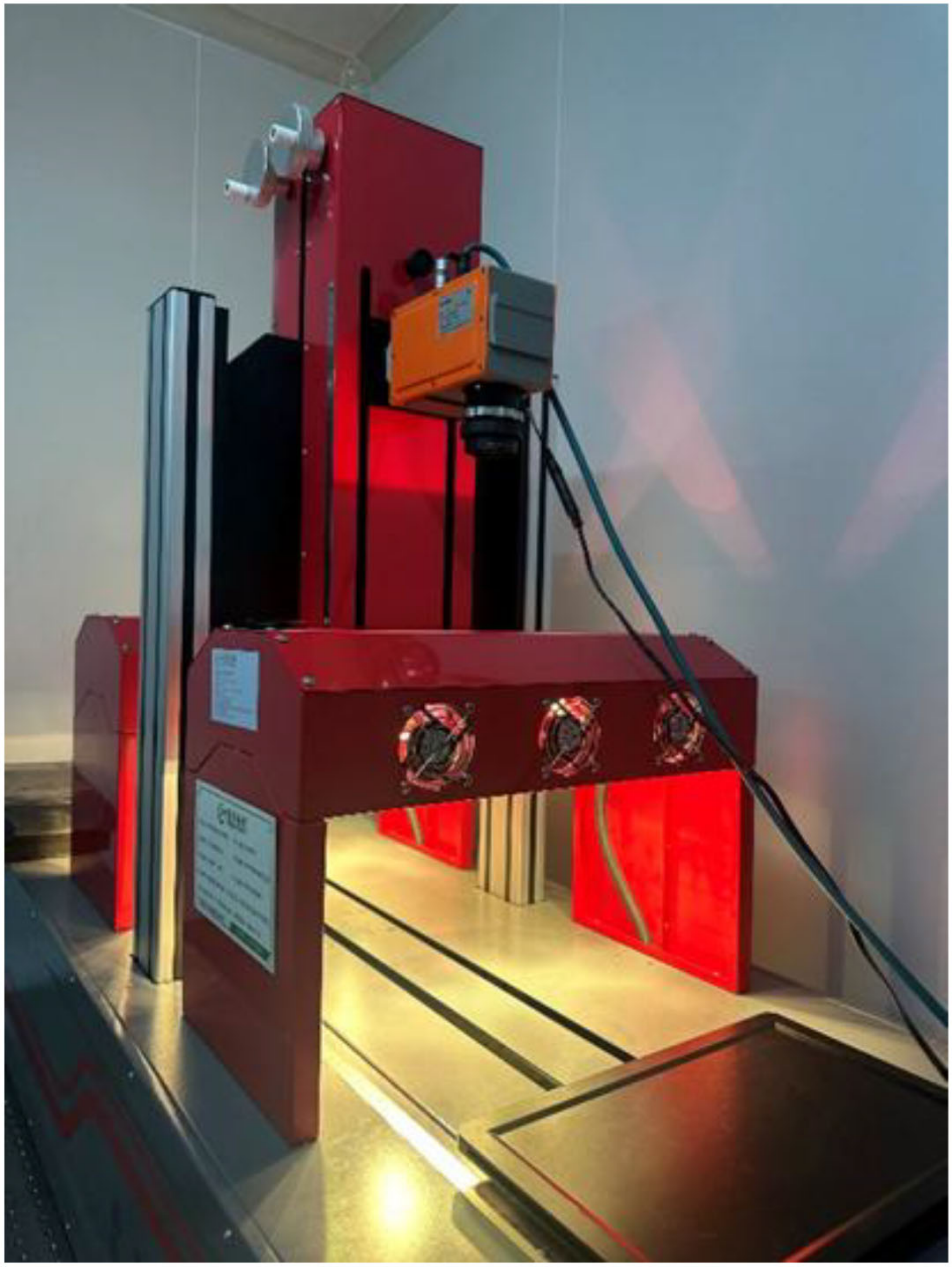
The RAP-HHIS-Q hyperspectral imaging system.

After each sampling dates, the samples were immediately sent to hyperspectral imaging lab to collect spectroscopy data. The RAP-HHIS-Q hyperspectral imaging system (GREENPHENO, WuHan, China) operating reflectance mode was used to acquire buckwheat hyperspectral reflectance data. The RAP-HHIS-Q hyperspectral imaging system is a push broom instrument equipped with a Specim FX17e hyperspectral camera sensor (Specim, Oulu, Finland), two 50-watt tungsten halogen lamps (GREENPHENO, WuHan, China), a conveyer belt and a computer (**Figure 2**). The Specim FX17e hyperspectral camera operates in the near-infrared (NIR) range, covering 900–1700 nm with a spectral resolution of 8 nm. It is equipped with a 640 × 512 pixel InGaAs sensor, providing high spatial resolution. The camera captures data across 224 spectral bands. During each measurement, approximately 100 buckwheat kernels from a single variety were evenly spread on a black background conveyor belt for scanning. Each measurement was repeated five times, and before each scan, a Spectralon^®^ white reference panel (Labsphere, Inc., North Sutton, NH, USA) was placed to facilitate subsequent reflectance calibration. The hyperspectral images were processed using ENVI 5.6 (Harris Geospatial Solutions, Inc., USA). The raw spectral data were imported into ENVI 5.6, and the Classification Tool was applied to distinguish buckwheat kernels from the background. After segmentation, the spectral reflectance of the extracted buckwheat grains was computed. To improve measurement accuracy, the reflectance values from five repeated scans were averaged for each variety.

### 2.3 WGAN-GP data augmentation

**Figure 3:**
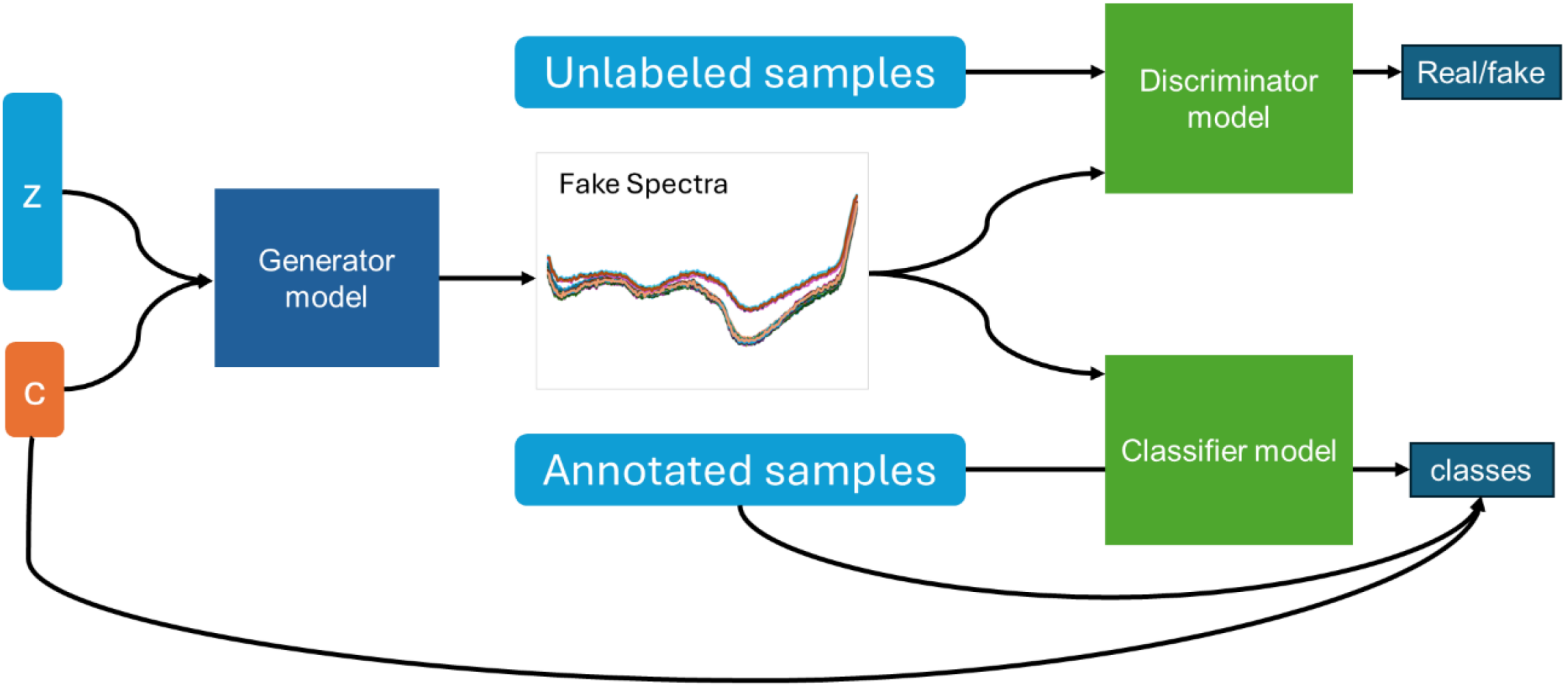
The architecture of WGAN-GP (z represents a random noise vector, c represents conditional label).

To enhance the performance of classification models, conditional WGAN-GP was employed as a data augmentation method for hyperspectral imaging spectroscopy data, effectively expanding the dataset. The conditional WGAN-GP is an advanced generative model that extends the traditional WGAN-GP by incorporating conditional constraints. It consists of three competing neural networks—the generator (G), discriminator (D), and classifier (C) (Goodfellow et al., 2020) (**Figure 3**). The network architectures are built using dense (fully connected) layers, with Leaky ReLU activation functions to enhance stability and prevent gradients vanishing. Weight initialization follows the Kaiming normal distribution (He et al., 2016) to improve convergence. The generator takes a random noise vector (z) as input, along with a conditional label (c) that represents different buckwheat maturity stages. It then synthesizes artificial spectral data that corresponds to the given label. The discriminator is responsible for distinguishing between real and generated data. It receives both actual spectral samples from the dataset and synthetic samples from the generator, then determines whether each input is authentic or artificially generated. The classifier ensures that the generated data not only appears realistic but also correctly matches the given conditional label. This additional network plays a crucial role in enforcing label consistency during generation. During training process, all three networks are trained simultaneously in an adversarial process, where the generator aims to fool both the discriminator and classifier, while the discriminator and classifier refine their ability to identify synthetic data and incorrect classifications. The hyperparameter setting of conditional WGAN-GP is shown in **Table 1**. The training process follows an adversarial framework with the Wasserstein distance as the optimization objective. The standard WGAN objective is given by the Wasserstein distance:

**Table 1:** The hyperparameter setting of synthetic hyperspectral reflectance data generation based on WGAN model.

Hyperparameter	Value
Batch Size	32
Learning rate	0.001
Noise dimension	30
Noise Type	Random
Number of features	186
Hidden layer size	512


$$\underset{G}{\text{min}}\underset{D}{\text{max}}\;E_{x\;\sim \;P_{r}}\left[D\left(x\right)\right]-\;E_{\tilde{x}\;\sim \;P_{g}}\left[D\left(\tilde{x}\right)\right]$$

$x\;\sim \;P_{r}$ represents real spectra samples from the dataset.

$\tilde{x}=G\left(z,\;c\right)\sim P_{g}$ represents generated spectral samples, where G takes random noise z and conditional label c as input.

D(x) and $D\left(\tilde{x}\right)$ represents the discriminator’s scores for real and fake samples.

To evaluate the quality and distributional characteristics of the synthetic spectral data generated by the conditional WGAN-GP model at different training epochs, this study applied Principal Component Analysis (PCA). PCA was conducted on both the original and generated datasets to project the high-dimensional spectral features into a two-dimensional space, allowing for direct comparison of their distributions. Specifically, this study extracted synthetic samples generated at various epochs (1,000; 2,000; 5,000; 10,000; and 20,000) and applied PCA trained on the original dataset to all samples to maintain consistency in the projection basis. At each selected epoch, synthetic samples were extracted and projected using PCA trained on the original dataset to ensure a consistent comparison basis. This allowed for visual inspection of the distributional similarity between generated and real spectra across maturity stages.

### 2.4 Classification models and assessments indicators

To better understand the impact of conditional WGAN-GP as a data augmentation method, this study compares classification performance between models trained with and without the generated spectral data. In this study four widely used ML classification models: PLS-LDA, KNN, RF, and SVM were used to classify different buckwheat maturity stages. PLS-LAD is a combination of PLSR with LDA working for classification tasks. PLS reduces dimensionality by extracting latent variables that maximize covariance between features and class labels, while LDA optimally separates classes. KNN is a simple, non-parametric classification algorithm that assigns a sample to the most common class among its K nearest neighbors in feature space. RF is an ensemble learning method that constructs multiple decision trees and aggregates their predictions for robust classification. Each tree is trained on a random subset of features and samples, improving generalization and reducing overfitting. SVM finds an optimal hyperplane to maximize the margin between different classes in a high-dimensional space. The hyperparameter setting of PLS-LDA, KNN, RF, and SVM are shown in **Table 2**. To evaluate the impact of generated artificial spectral data on classification task performance, the original training set is used as a baseline. First, three different random seeds were set to account for variability in data partitioning. For each seed, the dataset was split into training and testing sets (8:2) using stratified sampling without replacement, ensuring that each maturity stage was proportionally represented. In each split, ML models were trained in two phases: first using only the original training data and then using an augmented training set that included the generated synthetic spectra. During training, 5-fold cross-validation was applied on the training set to optimize model hyperparameters and prevent overfitting. The final model performance was evaluated on the corresponding held-out test set for each seed. The testing set was used to evaluate the performance of different ML models based on the Kappa coefficient, Accuracy, Precision, F1-score, Area Under the ROC Curve (AUC)and Confusion Matrices The calculation of Accuracy, Recall, and Precision was defined bellow:

**Table 2:** The average classification performance of four ML models on the original dataset.

Performance metrics	SVM	RF	KNN	PLS-LDA
Accuracy	92%	91%	89%	95%
Recall	91%	91%	89%	95%
Precision	92%	91%	89%	95%
F1-score	91%	90%	89%	95%
AUC	0.91	0.97	0.96	0.98
Kappa	0.88	0.87	0.85	0.93


$$Acc=\;\frac{TP+TN}{TP+FN+FP+TN}$$

$$Recall=\;\frac{TP}{TP+FN}$$

$$Precision=\;\frac{TP}{TP+FP}$$

$$F1-score=2*\;\frac{Precision*Recall}{Precision+Recall}$$

where TP, FN, FP, and TN are the true positive, false negative, false positive, true negative.

The conditional WGAN-GP and ML models in this study were implemented in Python 3.11.2, utilizing the scikit-learn library for machine learning algorithms and the PyTorch framework for building and training the conditional WGAN-GP. To assess whether the inclusion of synthetic data led to statistically significant performance improvements, we conducted a paired t-test comparing the model results with and without data augmentation across the three random seed splits. This helped determine whether the observed gains from synthetic data integration were consistent and reliable, beyond random variation.

## 3 Result

### 3.1 Buckwheat hyperspectral reflectance data

**Figure 4:**
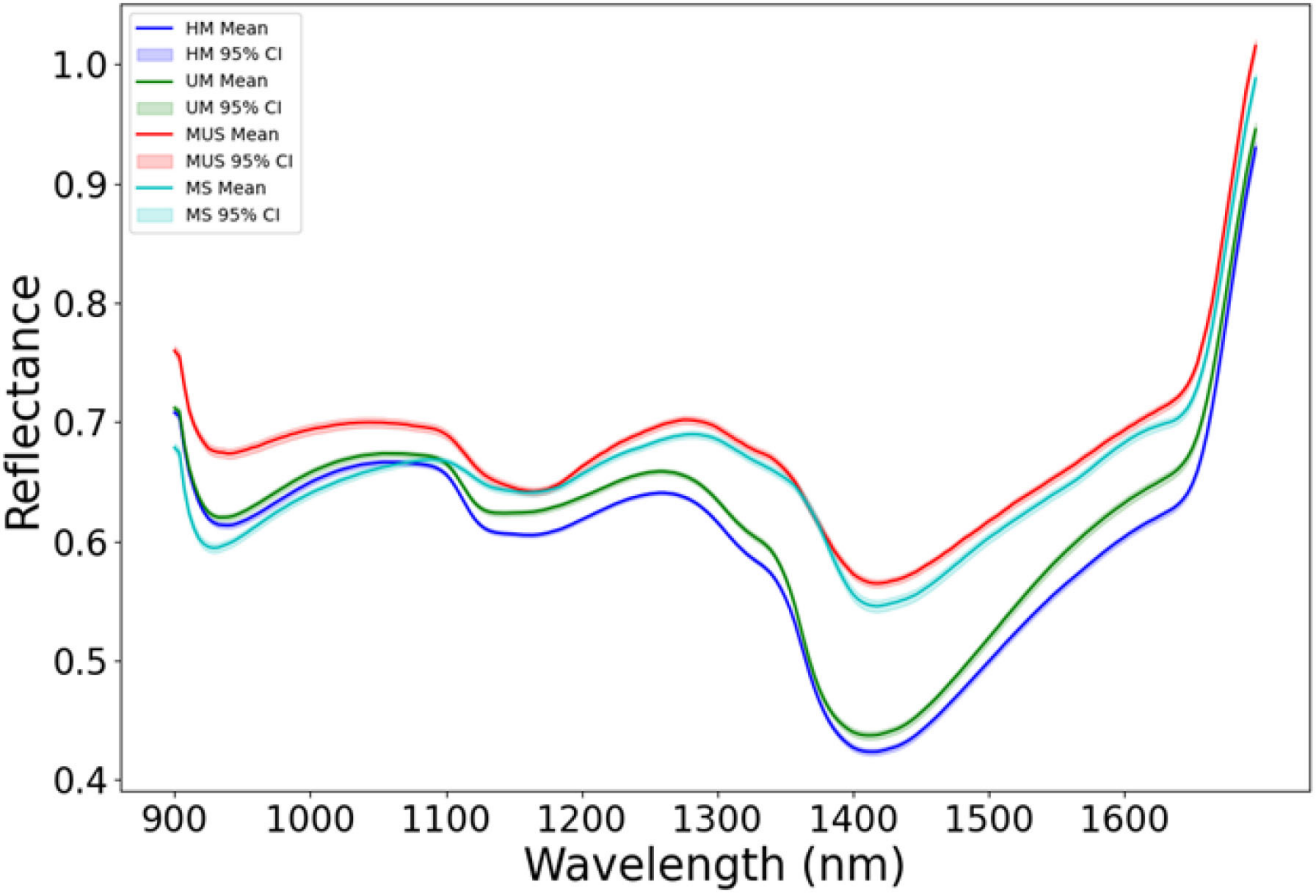
The average hyperspectral reflectance data of different buckwheat harvest periods (UM represents growth cycles 65 days, HM represents growth cycles 75 days, MS represents growth cycles 85 days with husks, MUS represents growth cycles 85 days and harvest stage dehulled).

In this study, the buckwheat maturity stages were divided into four categories including: UM represents growth cycles 65 days, HM represents growth cycles 75 days, MS represents growth cycles 85 days with husks, MUS represents growth cycles 85 days and harvest stage dehulled. **Figure 4** shows the average hyperspectral reflectance data of different buckwheat maturity stages. In the wavelength range of 900–1700 nm, the reflectance curves for all four harvest stages show similar overall trends, with strong absorption in the 1400 nm and high reflectance in the 1600 nm. The strong absorption around 1400 nm are related to the water content and caused by O-H stretching overtones (Xin et al., 2024). The moderate absorption in the 900–1000 nm are correlated with C-H and O-H bond vibrations in organic matter in buckwheat (Platov et al., 2021). A local reflectance peak in 1200–1300 nm, are related to structural carbohydrates (cellulose, hemicellulose) in the buckwheat. After 1600 nm, the reflectance value increases rapidly due to the scattering from starch and proteins. MUS (85 days, dehulled) shows the highest reflectance across 900–1700 nm, due to the absence of husk, which increases the exposure of the inner kernel structure. MS (85 days, with husks) has slightly lower reflectance than MUS, indicating that husk presence affects spectral properties. HM (75 days) and UM (65 days) have lower reflectance, especially in the 1400–1500 nm range, indicating early harvest buckwheat may have higher water content, leading to stronger absorption.

### 3.2 Synthetic spectral data generated from conditional WGAN-GP

**Figure 5:**
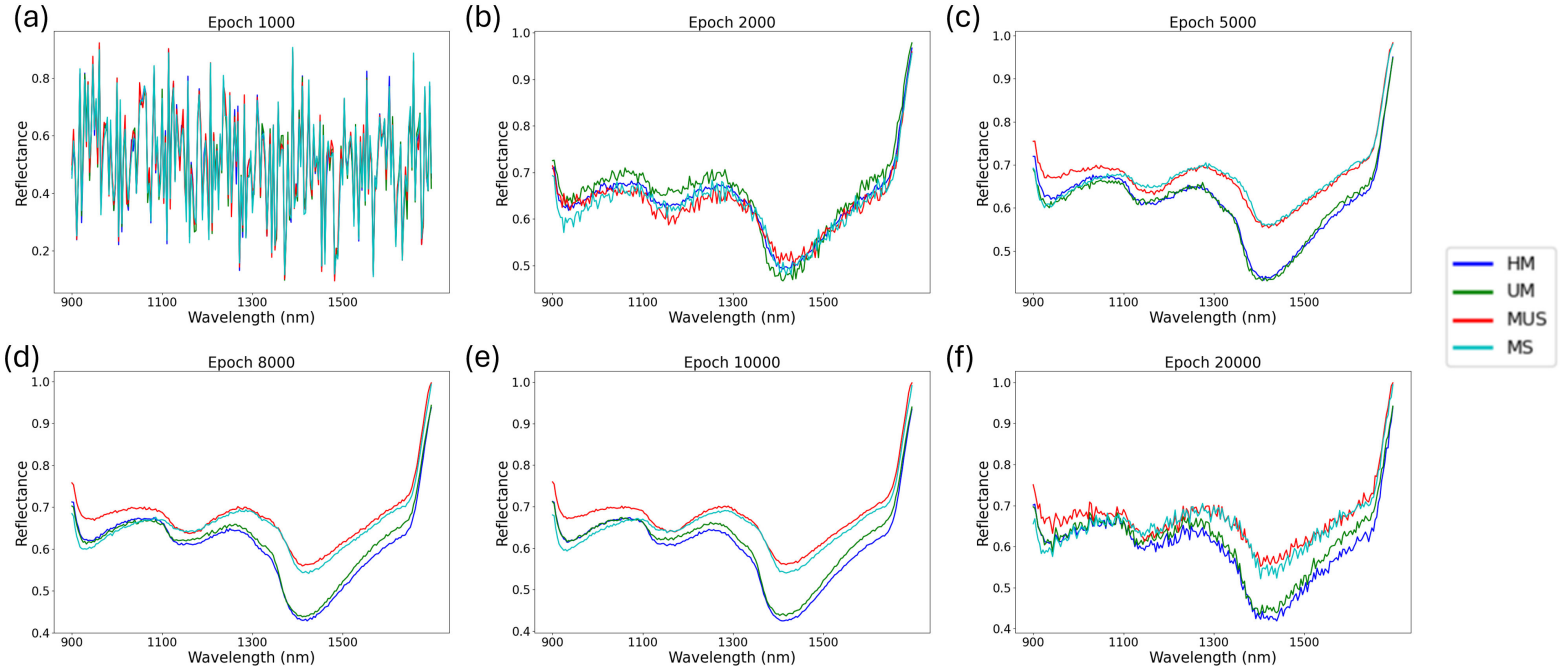
Spectrum of different buckwheat maturity stages generated by conditional WGAN-GP at epoch: 1000 **(a)**, 2000 **(b)**, 5000 **(c)**, 8000 **(d)**, 10,000 **(e)** and 20,000 **(f)** (UM represents growth cycles 65 days, HM represents growth cycles 75 days, MS represents growth cycles 85 days with husks, MUS represents growth cycles 85 days and harvest stage dehulled).

Conditional WGAN-GP has been trained for different epoch including: 1000, 2000, 5000, 8000, 10,000 and 20,000. This study generates 100 hyperspectral reflectance data for each buckwheat maturity stage for different training epoch (**Figure 5**). When the epoch is 1000, the generated artificial hyperspectral reflectance data is random noise (**Figure 5a**). When epoch is 2000, the generated artificial hyperspectral reflectance data show a spectral curve of buckwheat, while the generated data cannot show the characteristic of the difference maturity stages. As the number of epochs grows to 5000, the generated artificial hyperspectral reflectance data is gradually smoother and show the characteristic of the MUS between other maturity stages (**Figure 5c**). When the epoch is 10,000, the generated hyperspectral artificial reflectance data become smooth and show the characteristics of different maturity stages (**Figure 5e**). After 10,000 epochs, the generated artificial hyperspectral reflectance data become noise, suggesting potential overfitting and instability at this stage (**Figure 5f**).

**Figure 6:**
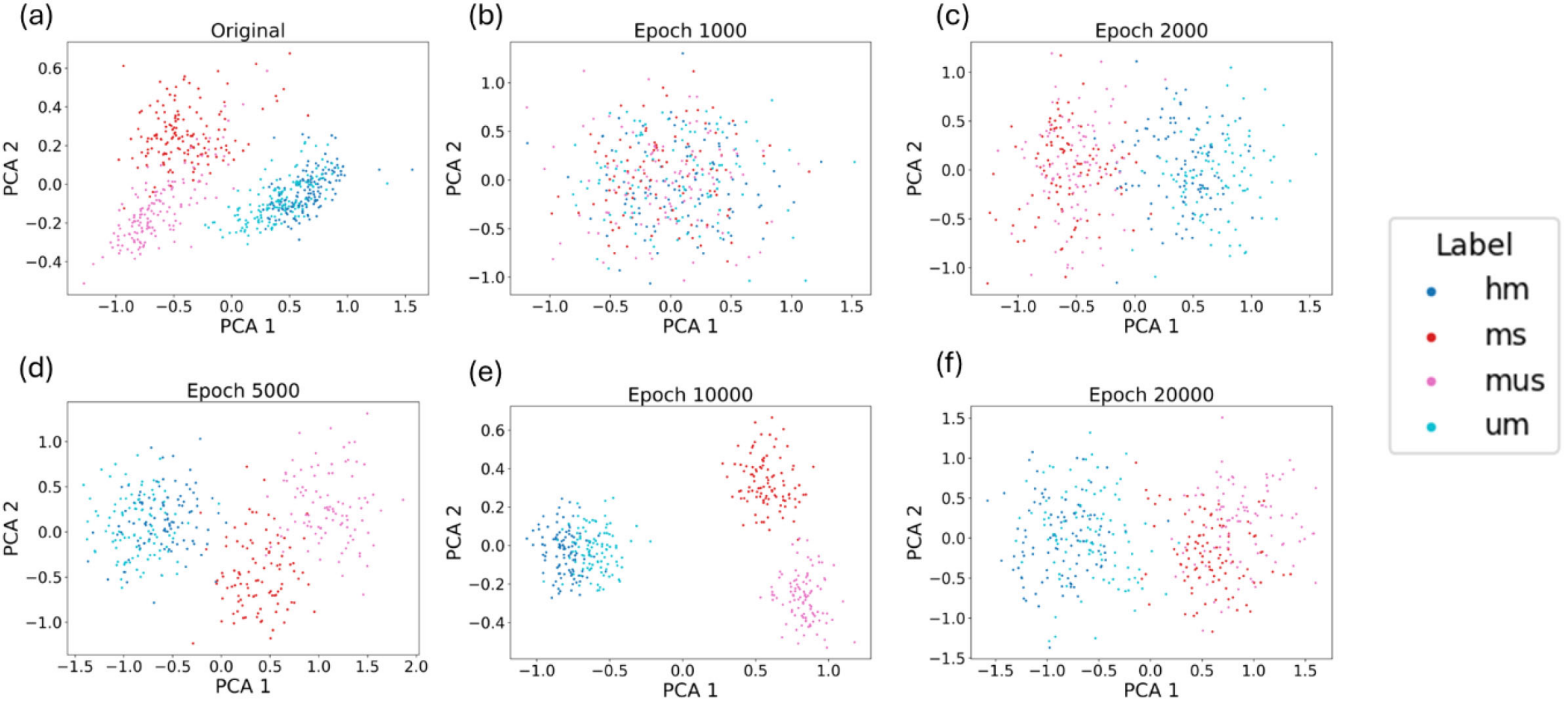
PCA visualization comparing the spectra of different buckwheat maturity stages from the original dataset **(a)** and those generated by conditional WGAN-GP at various training epochs: **(b)** 1,000, **(c)** 2,000, **(d)** 5,000, **(e)** 10,000, and **(f)** 20,000 (UM represents growth cycles 65 days, HM represents growth cycles 75 days, MS represents growth cycles 85 days with husks, MUS represents growth cycles 85 days and harvest stage dehulled).

**Figure 6** presents the PCA visualization of spectral data for different buckwheat maturity stages, comparing the original dataset (**Figure 6a**) with synthetic samples generated by the conditional WGAN-GP at various training epochs (**Figures 6b–f**). In the original data (**Figure 6a**), PCA1 and PCA2 effectively separate most maturity stages, with the exception of UM and HM, which exhibit significant overlap. At epoch 1,000 (**Figure 6b**), the generated spectra show no clear clustering, indicating the model had not yet learned meaningful class distinctions. By epoch 2,000 (**Figure 6c**), the synthetic MS and MUS samples begin to form a distinguishable cluster from UM and HM, although separation within the MS/MUS and UM/HM pairs remains unclear. At epoch 5,000 (**Figure 6d**), this pattern persists, but with a noticeable trend toward separation between MS and MUS, suggesting the model is starting to capture finer class-level structure. At epoch 10,000 (**Figure 6e**), the synthetic data shows a distribution highly consistent with the original dataset, with clear separation between most classes except for UM and HM, mirroring the real-data overlap. However, at epoch 20,000 (**Figure 6f**), the clustering quality deteriorates, with class boundaries becoming less defined. This suggests that the generator may have overfit or collapsed to less diverse outputs, leading to diminished representational fidelity. These results support the choice of 10,000 epochs as the optimal point for generating realistic and class-distinct synthetic spectra.

### 3.3 Classification result

**Figure 7:**
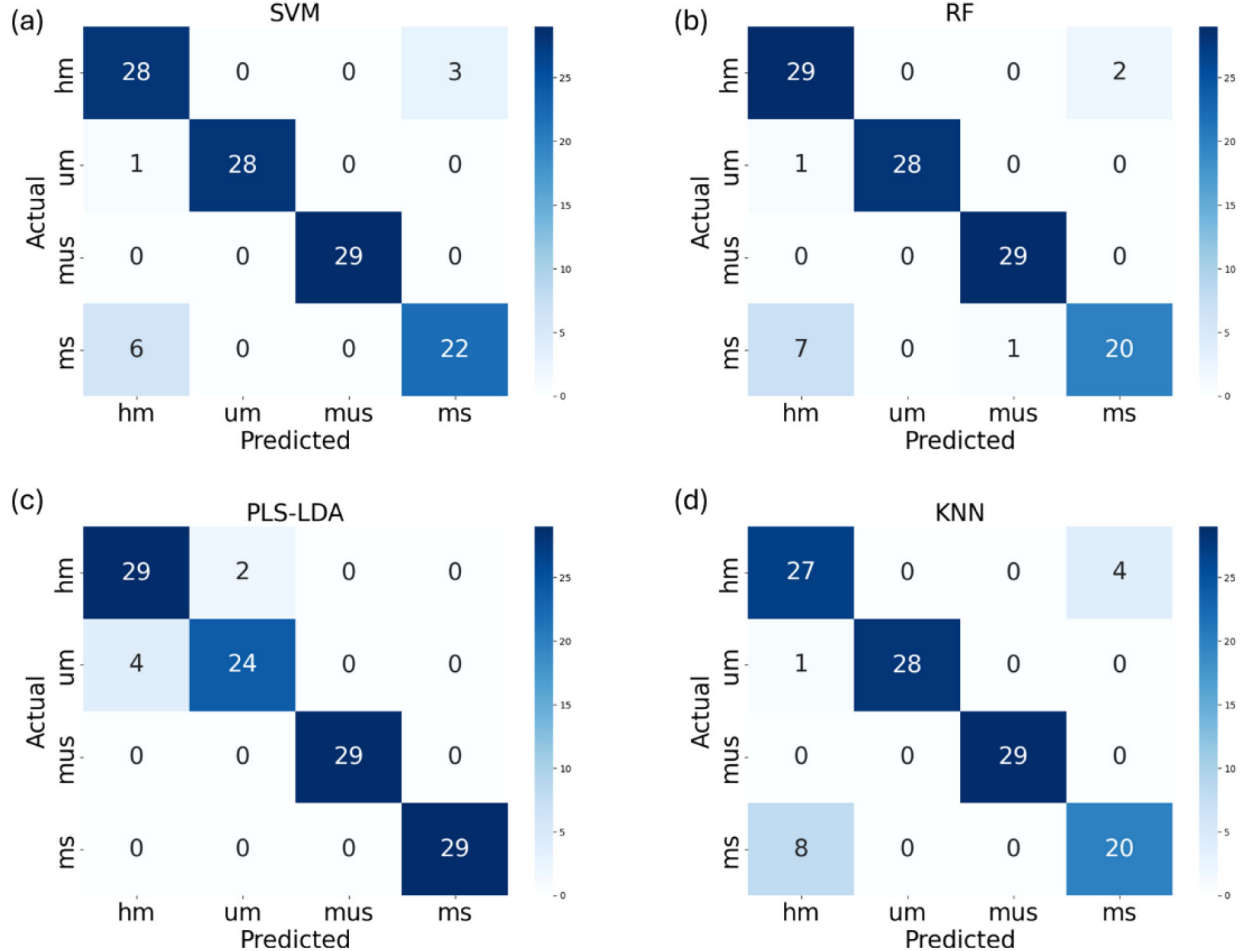
The confusion matrix of SVM **(a)**, RF **(b)**, PLS-LDA **(c)** and KNN **(d)** models’ classification performance on the original dataset (UM represents growth cycles 65 days, HM represents growth cycles 75 days, MS represents growth cycles 85 days with husks, MUS represents growth cycles 85 days and harvest stage dehulled).

This study evaluates the performance of four ML models for buckwheat maturity stages classification task. **Table 2** shows the classification performance of four ML models on the original dataset. Four ML models were trained on the original training dataset and validated the classification performance on the test dataset. **Table 2** shows the PLS-LDA has the best classification performance with accuracy of 95% and kappa coefficient of 0.93 on the test dataset. Compared with PLS-LDA, SVM and RF shows moderate classification performance with accuracy of 92% and 91%, kappa coefficient of 0.88 and 0.87, respectively. However, the classification performance of KNN was lower than other ML models, with accuracy of 89% and kappa coefficient of 0.85. The confusion matrix of four ML models’ classification performance on the original dataset is shown in **Figure 7**.

**Figure 8:**
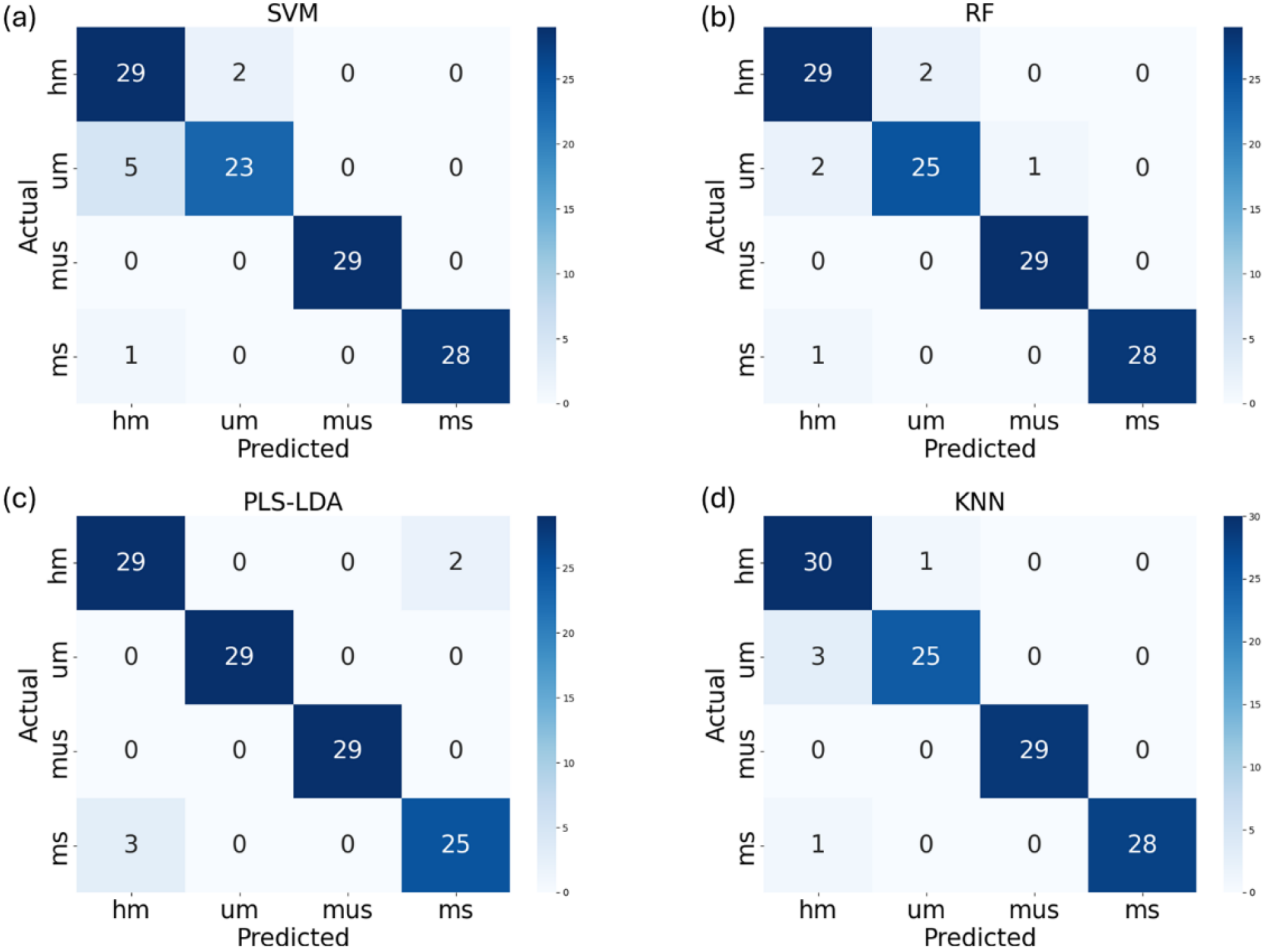
The confusion matrix of SVM **(a)**, RF **(b)**, PLS-LDA **(c)** and KNN **(d)** models’ classification performance on the original + synthetic dataset (UM represents growth cycles 65 days, HM represents growth cycles 75 days, MS represents growth cycles 85 days with husks, MUS represents growth cycles 85 days and harvest stage dehulled).

To evaluate the impact of artificially generated hyperspectral reflectance data on the performance of four common ML models. The models were trained using the original training dataset augmented with the generated spectral dataset. Based on the results in Section 3.2, the generated spectra dataset corresponding to an epoch of 10,000 in the conditional WGAN-GP training process was selected for use. In this dataset, 100 artificial spectra were generated for each maturity label, resulting in a total of 400 additional artificial samples incorporated into the original training dataset. **Table 3** shows the classification performance of four ML models on the original + synthetic dataset. After adding generated artificial hyperspectral reflectance data, PLS-LDA, SVM, RF and KNN resulted in higher accuracy compared to training solely on original training set with classification accuracy of 96%, 93%, 97% and 95%, respectively. Paired t-tests were conducted to determine whether the inclusion of synthetic spectra led to statistically significant performance improvements across different classification models. Results showed that both RF and KNN benefited significantly from data augmentation (p = 0.0004), indicating that synthetic spectra can improve these models’ performance. In contrast, SVM and PLS-LDA did not show statistically significant improvements (p > 0.05), possibly due to the saturation of performance using only original data. These findings suggest that the benefit of synthetic data may vary by model type. The best classification performance is achieved by RF model with accuracy of 97% and kappa coefficient of 0.94. The confusion matrix of four ML models’ classification performance on the original + synthetic dataset is shown in **Figure 8**.

**Table 3:** The average classification performance of four ML models on the original + synthetic dataset.

Performance metrics	SVM	RF	KNN	PLS-LDA
Accuracy	93%	97%	95%	96%
Recall	93%	97%	95%	96%
Precision	94%	97%	95%	96%
F1-score	93%	95%	96%	96%
AUC	0.99	0.99	0.98	0.99
Kappa	0.91	0.94	0.93	0.94

## 4 Discussion

This study evaluates the classification performance of four common ML models (PLS-LDA, SVM, RF, and KNN) for identifying buckwheat maturity stages based on hyperspectral reflectance data. The results indicate that PLS-LDA achieved the highest classification accuracy (96%) and kappa coefficient (0.94) on the test dataset, suggesting its strong ability to capture spectral variations among different maturity stages. Compared with previous study that apply machine learning models to classify red mountain buckwheat harvest period based on spectra data in NIR, this study’s result align with findings that spectra data in NIR range have potential to classify buckwheat harvest period in a rapid and non-destructive way (Xin et al., 2024). In addition, SVM and RF exhibited moderate classification performance when processing high-dimensional spectral data. Studies such as Lyu et al. (2024) have reported that SVM and RF tend to perform well when trained based high-dimensional features in both of regression and classification tasks.

To further improve classification performance, this study incorporated artificially generated hyperspectral reflectance data using a conditional WGAN-GP model. In contrast to conventional GAN architecture, this study incorporates an additional classifier within the network. By conditioning generated samples on specific class labels, the classifier enhances both their quality and diversity. To optimize the balance between data fidelity and computational efficiency, the conditional WGAN-GP was trained across multiple epochs: 1000, 2000, 8000, 10,000, and 20,000. As discussed in Section 3.2, visual analysis revealed that synthetic spectra produced at 10,000 epochs displayed smooth spectral curves and distinct characteristics corresponding to different buckwheat maturity stages. In addition, we conducted a PCA-based comparative analysis (**Figure 6**) to examine the distributional similarity between real and synthetic samples across different epochs. The PCA results supported our visual observations, indicating that the model achieved optimal stage-wise separability at 10,000 epochs, while overfitting effects emerged at 20,000 epochs. While this study primarily relied on visual inspection and PCA-based analysis to assess the quality of synthetic spectral data, future research should explore the use of quantitative evaluation metrics to more rigorously assess the realism of generated samples. In addition, future research should investigate automated or quantitative strategies for hyperparameter optimization to enhance the robustness and reproducibility of GAN-based data augmentation. Metrics such as the Fréchet Inception Distance (FID) and Kernel Inception Distance (KID), although originally developed for image and signal generation tasks, have been widely adopted in generative modeling due to their ability to capture distributional differences between real and synthetic data.

Most previous studies have applied GANs to generate RGB images or NIR spectral data without labels in the agricultural field (Lu et al., 2022; Jiang et al., 2023). In contrast, this study utilizes conditional WGAN-GP to generate hyperspectral reflectance data with different labels, enabling the generation of spectra specific to each maturity stage. This helps enhance data diversity, improve model generalization, and address the limitations of insufficient real spectral samples in agricultural classification tasks. The augmented dataset, which included 400 synthetic spectra data, resulted in improved classification accuracy for RF, and KNN, demonstrating the effectiveness of data augmentation in refining decision boundaries and reducing model overfitting. RF showed the best performance after augmentation, achieving an accuracy of 97% and a kappa coefficient of 0.94. However, SVM and PLS-LDA did not show any improvement, indicating that the synthetic data might not have provided additional information beyond what was already captured by the model. The generalizability of WGAN-GP across different seasons, cultivars, or environmental conditions remains an open question. The data used in this study were collected under specific conditions, and further study needs to confirm whether the model and the synthetic data it produces remain effective under varied field scenarios.

## 5 Conclusion

In this study, four maturity stages of buckwheat were investigated using hyperspectral reflectance data collected from 146 varieties in an experimental field. The stages included irrigation (65 days), green-ripe (75 days), harvest with husks (85 days), and harvest dehulled (85 days), with spectral data spanning 900–1700 nm. To address data scarcity and improve classification performance, conditional WGAN-GP was applied to generate synthetic hyperspectral samples for different maturity stages. Four machine learning classifiers—SVM, RF, KNN, and PLS-LDA—were evaluated. Using the original dataset, PLS-LDA achieved the highest accuracy (95%) and kappa (0.93). When trained on combined real and synthetic data, RF and KNN models showed performance improvements, with RF achieving the best overall accuracy (97%) and kappa (0.94). These results demonstrate that synthetic data augmentation via conditional WGAN-GP can effectively enhance classification accuracy for buckwheat maturity stages. However, this study is limited by its focus on a single crop and location, which may affect the generalizability of the models to other crops or environmental conditions. Additionally, generating synthetic data using WGAN-GP incurs computational costs that should be considered for practical applications. Future work will involve testing the approach on multi-site and multi-year datasets to improve robustness, as well as integrating UAV-based hyperspectral imaging to enable high-throughput and real-time crop monitoring.

## Data Availability

The raw data supporting the conclusions of this article will be made available by the authors, without undue reservation.
