# Correction: Enhancing buckwheat maturity classification with generative adversarial networks for spectroscopy data augmentation

**DOI:** 10.3389/fpls.2025.1757122

**Published:** 2025-12-18

**Authors:** Huihui Wang, Xiaoxue Che, Jiaxuan Nan, Yuyuan Miao, Yaqi Wang, Wuping Zhang, Fuzhong Li, Jiwan Han

**Affiliations:** Software College, Shanxi Agricultural University, Taigu, Shanxi, China

**Keywords:** buckwheat, spectroscopy, machine learning, generative adversarial networks, NIR, precision agriculture

An incorrect **Funding** statement was provided. The original **Funding** statement stated, “the National Natural Science Foundation of China (NSFC)(U21A20216) and Shanxi Key Project (2022ZDYF108)”. The correct funder is “China Agriculture Research System of MOF and MARA(CARS-07); the grand science and technology special project in Shanxi Province (202101140601027)”. The correct **Funding** statement reads:

“The research was funded by China Agriculture Research System of MOF and MARA(CARS-07), and the grand science and technology special project in Shanxi Province (202101140601027)”.

In the **Acknowledgements**, the contributions of Mingyang Zhang, Dake Guo, and Zhaoxia Sun to this research were erroneously omitted. The original **Acknowledgements** section only stated: “We acknowledge the valuable contribution of staff and students of Shanxi Agricultural University for collecting the field samples.” The correct **Acknowledgements** section reads, “We acknowledge the valuable contribution of staff and students of Shanxi Agricultural University for collecting the field samples. We also express our sincere gratitude to Mr. Mingyang Zhang and Mr. Dake Guo for their participation in buckwheat sample collection and hyperspectral data acquisition, which laid a solid foundation for the experimental data of this study; we thank Prof. Zhaoxia Sun for her contributions to the optimization of research methodology and critical revision of the manuscript, which improved the scientific rigor and clarity of the work”.

The original version of this article has been updated.

